# Low muscle mass, malnutrition, sarcopenia, and associations with survival in adults with cancer in the UK Biobank cohort

**DOI:** 10.1002/jcsm.13256

**Published:** 2023-05-22

**Authors:** Nicole Kiss, Carla M. Prado, Robin M. Daly, Linda Denehy, Lara Edbrooke, Brenton J. Baguley, Steve F. Fraser, Abbas Khosravi, Gavin Abbott

**Affiliations:** ^1^ Institute for Physical Activity and Nutrition Deakin University Geelong Australia; ^2^ Department of Health Services Research Peter MacCallum Cancer Centre Melbourne Australia; ^3^ Department of Agricultural, Food and Nutrition Science University of Alberta Edmonton Canada; ^4^ Melbourne School of Health Sciences University of Melbourne Parkville Australia; ^5^ Department of Physiotherapy University of Melbourne Parkville Australia; ^6^ Institute for Intelligent Systems Research and Innovation Deakin University Geelong Australia

**Keywords:** Cancer, Low muscle mass, Malnutrition, Mortality, Sarcopenia, UK Biobank

## Abstract

**Background:**

Low muscle mass (MM) is a common component of cancer‐related malnutrition and sarcopenia, conditions that are all independently associated with an increased risk of mortality. This study aimed to (1) compare the prevalence of low MM, malnutrition, and sarcopenia and their association with survival in adults with cancer from the UK Biobank and (2) explore the influence of different allometric scaling (height [m^2^] or body mass index [BMI]) on low MM estimates.

**Methods:**

Participants in the UK Biobank with a cancer diagnosis within 2 years of the baseline assessment were identified. Low MM was estimated by appendicular lean soft tissue (ALST) from bioelectrical impedance analysis derived fat‐free mass. Malnutrition was determined using the Global Leadership in Malnutrition criteria. Sarcopenia was defined using the European Working Group on Sarcopenia in Older People criteria (version 2). All‐cause mortality was determined from linked national mortality records. Cox‐proportional hazards models were fitted to estimate the effect of low MM, malnutrition, and sarcopenia on all‐cause mortality.

**Results:**

In total, 4122 adults with cancer (59.8 ± 7.1 years; 49.2% male) were included. Prevalence of low MM (8.0% vs. 1.7%), malnutrition (11.2% vs. 6.2%), and sarcopenia (1.4% vs. 0.2%) was higher when MM was adjusted using ALST/BMI compared with ALST/height^2^, respectively. Low MM using ALST/BMI identified more cases in participants with obesity (low MM 56.3% vs. 0%; malnutrition 50% vs. 18.5%; sarcopenia 50% vs. 0%). During a median 11.2 (interquartile range: 10.2, 12.0) years of follow up, 901 (21.7%) of the 4122 participants died, and of these, 744 (82.6%) deaths were cancer‐specific All conditions were associated with a higher hazard of mortality using either method of MM adjustment: low MM (ALST/height^2^: HR 1.9 [95% CI 1.3, 2.8], *P* = 0.001; ALST/BMI: HR 1.3 [95% CI 1.1, 1.7], *P* = 0.005; malnutrition (ALST/height^2^: HR 2.5 [95% CI 1.1, 1.7], *P* = 0.005; ALST/BMI: HR 1.3 [95% CI 1.1, 1.7], *P* = 0.005; sarcopenia (ALST/height^2^: HR 2.9 [95% CI 1.3, 6.5], *P* = 0.013; ALST/BMI: HR 1.6 [95% CI 1.0, 2.4], *P* = 0.037).

**Conclusions:**

In adults with cancer, malnutrition was more common than low MM or sarcopenia, although all conditions were associated with a higher mortality risk, regardless of the method of adjusting for MM. In contrast, adjustment of low MM for BMI identified more cases of low MM, malnutrition, and sarcopenia overall and in participants with obesity compared with height adjustment, suggesting it is the preferred adjustment.

## Introduction

Muscle loss is a common consequence of a cancer diagnosis or treatment due to the metabolic effects of the tumour, systemic inflammation, and treatment‐related gastrointestinal toxicities, affecting nutritional intake and other regulatory functions.^1^ Low muscle mass is estimated to be present in 25% to 60% of people at the time of cancer diagnosis, with a higher prevalence in certain cancer types such as lung and head and neck cancers.^2^, ^3^ Furthermore, muscle loss in people with cancer is known to occur at rates up to 10‐fold greater than those observed during aging.^4^ Low muscle mass in people with cancer is independently associated with up to a two‐fold increased risk of mortality.^5^, ^6^

Low muscle mass is also an integral component of the diagnosis of malnutrition and is one of three phenotypic criteria considered in the Global Leadership Initiative on Malnutrition (GLIM) criteria.^7^, ^8^ The GLIM criteria were developed jointly by global clinical nutrition societies and are the proposed global consensus criteria for diagnosing malnutrition in clinical populations.^8^ Additionally, despite a lack of global agreement on a definition of sarcopenia, low muscle mass is a key component of most definitions proposed by various professional bodies and societies.^9^, ^10^, ^11^, ^12^ Similar to low muscle mass alone, malnutrition, and sarcopenia are both independently associated with reduced survival in people with cancer.^2^, ^13^, ^14^

As low muscle mass is a common underlying component of disease‐related malnutrition and sarcopenia, an accurate assessment of muscle mass (or its related compartments—lean soft tissue and fat‐free mass) is critical. Muscle mass is strongly related with overall body size, and thus, adjustments for body size by factors such as height or body mass index (BMI) are often used as a standardized method of scaling.^9^ However, the optimal method of adjustment for muscle mass remains a subject of continued debate, with the suggestion that preferred methods may differ across various clinical populations,^9^ with no consensus on the method in adults with cancer.

Current research in cancer populations has predominantly adjusted muscle mass for height in metres squared,^2^, ^6^ although recent evidence has questioned whether this is the best allometric scaling approach due to wide variation in the proportion of muscle mass observed across all BMI categories.^15^ On a whole‐body level, there is limited understanding of whether adjustment of muscle mass for BMI compared with height affects the proportion of patients with cancer classified as having low muscle mass, and subsequently the prevalence of low muscle mass, malnutrition or sarcopenia. Low muscle mass, lean soft tissue or fat free mass (FFM), assessed by computed tomography (CT), dual energy X‐ray absorptiometry (DXA) or bioelectrical impedance analysis (BIA), and adjusted for height in metres squared has been shown to be independently associated with an increased risk of mortality in adults with cancer.^2^, ^5^, ^16^, ^17^ Less is understood regarding the association between BMI‐adjusted low muscle mass and survival in cancer populations, or whether the method of adjustment alters the strength of the association with mortality. Improving our understanding of which method of muscle mass scaling (e.g., height or BMI) may be better related to adverse clinical outcomes can improve risk stratification and prognostication of patients with low muscle mass or its related conditions. Therefore, the primary objective of this study was to compare the prevalence of low muscle mass, malnutrition, and sarcopenia and their association with survival in adults with cancer in the UK Biobank study. The secondary objective was to explore the influence of the most common methods of allometric scaling (height in metres squared and BMI) on the estimated prevalence of low muscle mass, malnutrition, and sarcopenia and their association with survival.

## Methods

### Study population

The UK Biobank is a population‐based prospective epidemiological study including human health data from over 500 000 adults aged 37 to 73 years from the United Kingdom.^18^ Participants were recruited for their baseline assessment at 22 centres across England, Wales, and Scotland between 2006 and 2010. The baseline assessment involved completion of a touch screen questionnaire (sociodemographic and lifestyle factors), physical measurements, blood, and urine samples.^18^ All participants with a cancer diagnosed within 2 years of their baseline assessment, excluding non‐melanoma skin cancer, were included in this study. The timeframe of 2 years was chosen to capture varying durations of treatment across different cancer types following a cancer diagnosis, and the potential for malnutrition and sarcopenia to persist beyond treatment.^19^ Some questionnaires and measurements used within the UK Biobank differ from those commonly used within current criteria and definitions for malnutrition and sarcopenia.^18^ Therefore, the criteria and definitions used in this study were adapted (Table 1) according to data available for the UK Biobank participants and as described in previous UK Biobank publications.^20^, ^21^ This paper is reported according to the criteria in the Strengthening the Reporting of Observational Studies in Epidemiology guidelines.^22^

**Table 1:** Adaptation of malnutrition, low muscle mass, and sarcopenia criteria for the UK Biobank

Component	Original criteria	UK Biobank
Malnutrition^a^		
Weight loss	>5% in past 6 months or >10% beyond 6 months	Self‐reported: ‘Compared with 1 year ago, has your weight changed?’ Response: yes, lost weight = 1 or other = 0 (no, gained weight, do not know)
Low BMI	*Mild to moderate*: <20 kg/m^2^ if <70 years or <22 kg/m^2^ if ≥70 years *Severe*: <18.5 kg/m^2^ if <70 years or <20 kg/m^2^ if ≥70 years	*Mild to moderate*: <20 kg/m^2^ if < 70 years or <22 kg/m^2^ if ≥70 years *Severe*: <18.5 kg/m^2^ if <70 years or <20 kg/m^2^ if ≥70 years
Low muscle mass	*Mild to moderate* deficit by validated assessment methods *Severe* deficit by validated assessment methods	*Mild to moderate*: ALST/height^2^ < 6.15 kg/m^2^ (women) and <7.98 kg/m^2^ (male) OR ALST/BMI < 0.64 (women) and <0.94 (men) *Severe*: ALST/height^2^ < 5.30 kg/m^2^ (women) and <6.95 kg/m^2^ (male) OR ALST/BMI < 0.55 (women) and <0.84 (men)
Inflammation	Acute disease/injury or chronic‐disease related	CRP ≥5 mg/L
Sarcopenia^b^		
Low muscle strength	Handgrip strength <27 kg (men) and <16 kg (women)	Handgrip strength <27 kg (men) and <16 kg (women)
Low muscle mass	ALST/height^2^ < 7.0 kg/m^2^ (men) or <5.5 kg/m^2^ (women)	ALST/height^2^ < 5.30 kg/m^2^ (women) and <6.95 kg/m^2^ (men) OR ALST/BMI < 0.55 (women) and <0.84 (men)
Low physical performance	Gait speed <0.8 m/s	Self‐reported: ‘How would you describe your usual walking pace?’ Response: slow = 1, other = 0 (average, brisk, none of the above)

ALST, appendicular lean soft tissue; BMI, body mass index; CRP, C‐reactive protein.

^a^ Assessed using the Global Leadership Initiative on Malnutrition (GLIM) criteria.

^b^ Assessed using the European Working Group on Sarcopenia in Older People 2 (EWGSOP2) definition. Sex‐specific cut‐points for low ALST/height^2^ and low ALST/BMI specific to the UK Biobank cohort were derived from participants aged 45 years or less as the reference, based on two standard deviations below the sex‐specific mean.^25^

### Demographics and cancer diagnosis

Demographics (age and sex) were acquired from participants at the assessment centre. Ethnicity (White and other), smoking status (never, previous, and current) and frequency of alcohol intake (daily or almost daily, one to four times per week, one to three times per month, never or special occasions only), and non‐cancer co‐morbid illnesses were self‐reported in the touchscreen questionnaire. Townsend deprivation scores, a measure of economic deprivation, were determined prior to the UK Biobank assessment from participant postcodes based on the preceding national census.^23^ A higher Townsend index indicates a higher degree of deprivation, with the UK census in 2011 showing a mean Townsend score of −3.80 (SD 0.63) in areas within the lowest quintile of deprivation and 5.45 (SD 2.9) in the highest quintile of deprivation.^24^

Date of cancer diagnosis and type of cancer according to the International Classification of Diseases 10th revision (ICD‐10) were determined from data linkage to national cancer registries. Individual ICD‐10 cancer codes were grouped into 11 broader cancer types including bone and soft tissue, breast, central and peripheral nervous system, endocrine and thyroid, gastrointestinal, genitourinary, haematological, head and neck, lung and other thoracic, melanoma, and unknown primary. The difference (years) between the date of cancer diagnosis and date of baseline assessment determined time since cancer diagnosis for each participant.

### Assessment of muscle mass

Low muscle mass was assessed by appendicular lean soft tissue (ALST) estimated from BIA‐derived fat‐free mass (Tanita BC418MA, Tokyo, Japan). This was done using an equation developed by Dodds et al., using data from UK Biobank participants who at a later assessment underwent a total body DXA scan for the assessment of ALST. The equation is as follows: ALST (kg) = (0.958 × [appendicular FFM (kg)]) − (0.166 × S) − 0.308, with S taking the value 0 if female and 1 if male.^20^

Sex‐specific cut‐points for low ALST/height^2^ and low ALST/BMI specific to the UK Biobank cohort were derived from participants aged 45 years or less as the reference, based on two standard deviations below the sex‐specific mean as per previously reported methods.^25^ This resulted in the following cut‐points: (1) low ALST/height^2^: <6.95 kg/m^2^ for men; <5.30 kg/m^2^ for women and (2) low ALST/BMI: <0.84 for men; <0.55 for women. The GLIM malnutrition diagnosis distinguishes between mild to moderate and severe reduced muscle mass.^8^ Therefore, we also derived cut‐points for mild to moderately reduced ALST/height^2^ and ALST/BMI, based on one standard deviation below the mean using a similar approach to previous reports.^26^ This resulted in cut‐points for mild to moderate low ALST/height^2^ of <7.98 kg/m^2^ for men and <6.15 kg/m^2^ for women, and low ALST/BMI of <0.94 for men and <0.64 for women.

ALST is predominantly skeletal muscle, with a small amount of skin and connective tissue; however, for simplicity, the term muscle mass is used throughout this paper.

### Assessment of malnutrition

The presence of malnutrition was determined using the GLIM criteria.^8^ For a diagnosis of malnutrition, at least one of three phenotypic criteria and one of two etiological criteria must be present. Phenotypic criteria include unintentional weight loss, low BMI and reduced muscle mass. Measures used to assess weight loss and low BMI are described in Table 1. Reduced muscle mass was determined from estimated ALST as described earlier. All phenotypic criteria were used for malnutrition diagnosis. Etiologic criteria include reduced food intake or assimilation and presence of inflammation.^8^ Quantifiable data on food intake were only available for a sub‐set of UK Biobank participants; therefore, etiologic criteria were assessed using inflammation alone. Currently, cut‐points to designate the presence of inflammation are not specified for the GLIM criteria. In this study serum C‐reactive protein of 5 mg/L or higher was used to represent moderate inflammation.^27^

### Assessment of sarcopenia

The presence of probable sarcopenia and sarcopenia were defined using the European Working Group of Sarcopenia in Older People 2 (EWGSOP2) definition^9^ for individuals of all ages. Participants were considered to have probable sarcopenia if they had low handgrip strength alone and confirmed sarcopenia if they also had low muscle mass according to the cut‐points described in Table 1. Sarcopenia was considered to be severe if low handgrip strength, low muscle mass and low physical performance were all present.

#### Handgrip strength

Grip strength was measured using a Jamar J00105 hydraulic hand dynamometer by a trained research nurse. One measurement was taken for each arm in an upright seated position with 90° elbow flexion and the forearm placed on armrests in mid‐prone position. The maximum value of the two measurements was used to determine peak grip strength (kg).^9^

#### Physical performance

The UK Biobank does not include an objective measure of physical performance. Therefore, participants were considered to have poor physical performance using self‐reported walking pace as described in previous studies of sarcopenia in UK Biobank participants (Table S1).^20^, ^28^

### Mortality

All‐cause mortality was determined using data from national mortality records linked to the UK Biobank. Mortality was deemed to be cancer‐specific where the primary cause of death was attributed to cancer using ICD‐10 codes. Data were censored on 3 August 2020, 3 months after receipt of the data from the UK Biobank to ensure completeness of the data.

### Statistical analysis

Sociodemographic and clinical characteristics (age, sex, cancer diagnosis, time since cancer diagnosis, ethnicity, Townsend deprivation index, weight, BMI, smoking status, alcohol frequency, and number of long‐term conditions (0, 1, 2, 3, and ≥4) were summarized for all participants using mean (standard deviation) or median (interquartile range) for continuous variables and as a count (percentage) for categorical variables. The prevalence (count [percentage]) of all conditions (low muscle mass, malnutrition, and sarcopenia) were summarized for the whole sample and by cancer diagnosis. Cox‐proportional hazards models were fitted to estimate the association between each condition and all‐cause and cancer‐specific mortality. Models were adjusted for known confounders of muscle mass and mortality: age, sex, BMI (height‐adjusted measures only), time since cancer diagnosis, smoking status, deprivation score, alcohol use, and number of co‐morbid conditions. Associations are reported as hazard ratios (HR) with 95% confidence intervals (CI). Covariate‐adjusted Kaplan–Meier curves were constructed separately for all‐cause and cancer‐specific survival for each condition (low muscle mass, malnutrition, and sarcopenia) using the height (in m^2^) and BMI adjustment, with the exception of sarcopenia using the height adjustment for which unadjusted Kaplan–Meier curves were constructed due to the low number of cases. The strength of the evidence against the null hypothesis was interpreted as follows: *P* < 0.001 very strong evidence, *P* < 0.01 strong evidence, *P* < 0.05 moderate evidence, *P* < 0.1 weak evidence, and *P* > 0.1 insufficient evidence.^29^

## Results

**Figure 1:**
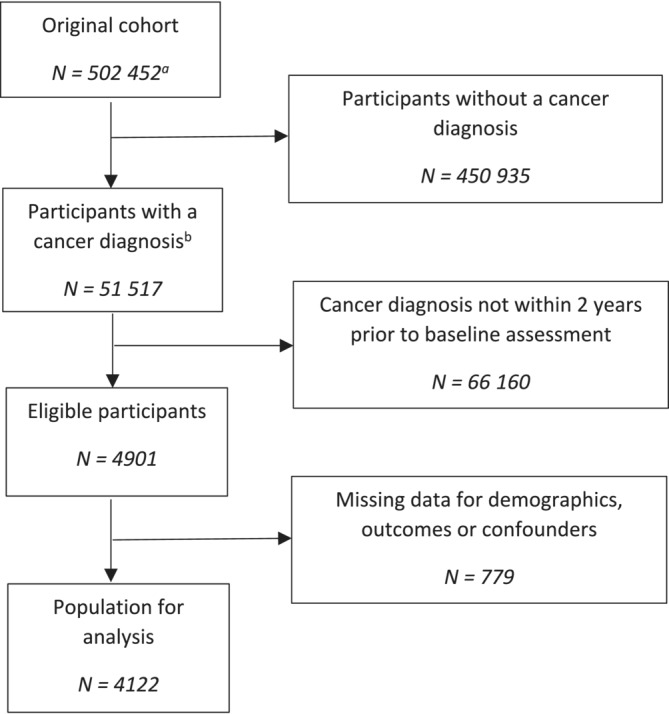
Study flow chart. ^a^Excludes participants' who have requested their data are removed from the UK Biobank. ^b^Excludes non‐melanoma skin cancer and benign tumours.

Overall, of the original 502 493 UK Biobank participants, 4122 (0.8%) were included in this analysis after excluding those without a cancer diagnosis within the 2 years prior to their baseline assessment and those with missing data (*n* = 779) for one or more of the conditions or covariates (Figure 1).

Participant characteristics are described in Table 2. The most common cancer diagnoses were genitourinary (28.9%) and breast (26.1%), followed by gastrointestinal (14.7%) cancer. Almost 70% of participants were under 65 years of age, 68% had overweight or obesity and 65% had at least one long‐term condition in addition to their cancer diagnosis.

**Table 2:** Participant characteristics (*N* = 4122)

Characteristic	Full sample (*n* = 4122)	Men (*n* = 2031)	Women (*n* = 2091)
	Mean ± SD or *n* (%)	Mean ± SD or *n* (%)	Mean ± SD or *n* (%)
Age (years)	59.8 ± 7.1	61.4 ± 6.4	58.3 ± 7.5
Age category (years)			
<65	2842 (68.9)	1256 (61.8)	1586 (75.8)
≥65	1280 (31.1)	775 (38.2)	505 (24.2)
Cancer diagnosis			
Genitourinary	1192 (28.9)	1138 (56.0)	54 (2.6)
Breast	1075 (26.1)	6 (0.3)	1069 (51.1)
Gastrointestinal	607 (14.7)	371 (18.3)	236 (11.3)
Haematological	311 (7.5)	185 (9.1)	126 (6.0)
Gynaecological	298 (7.2)	‐	298 (14.3)
Melanoma	288 (7.0)	132 (6.5)	156 (7.5)
Head and neck	118 (2.9)	81 (4.0)	37 (1.8)
Lung and other thoracic	82 (2.0)	50 (2.4)	32 (1.5)
Endocrine and thyroid	56 (1.4)	14 (0.7)	42 (2.0)
Central and peripheral nervous system	37 (0.9)	16 (0.8)	21 (1.0)
Bone and soft tissue	31 (0.7)	24 (1.2)	7 (0.3)
Unknown primary	27 (0.7)	14 (0.7)	13 (0.6)
Time since cancer diagnosis (years), median [IQR]	1.1 [0.7, 1.5]	1.1 [0.6, 1.5]	1.1 [0.7, 1.6]
Ethnic background			
White	3984 (96.6)	1965 (96.7)	2019 (96.6)
Non‐white	122 (3.0)	54 (2.7)	68 (3.3)
Prefer not to say	16 (0.4)	12 (0.6)	4 (0.1)
Weight (kg)	78.6 ± 15.7	84.9 ± 14.1	72.4 ± 14.6
Body mass index (kg/m^2^)	27.6 ± 4.8	27.7 ± 4.1	27.4 ± 5.4
Body mass index category (kg/m^2^)			
<18.5	18 (0.4)	9 (0.5)	9 (0.4)
18.5–24.9	1298 (31.5)	519 (24.5)	779 (37.3)
25.0–29.9	1776 (43.1)	1011 (49.8)	765 (36.6)
≥30	1030 (25.0)	492 (24.2)	538 (25.7)
Smoking status			
Never	2035 (49.4)	884 (43.5)	1151 (55.0)
Previous	1720 (41.7)	951 (46.8)	769 (36.8)
Current	367 (8.9)	196 (9.7)	171 (8.2)
Alcohol frequency			
Daily or almost daily	816 (19.8)	490 (24.1)	326 (15.6)
1–4 times per week	1911 (46.4)	1000 (49.2)	911 (43.6)
1–3 times per month	484 (11.8)	186 (9.2)	298 (14.2)
Occasional or never	911 (22.0)	355 (17.5)	556 (26.6)
Townsend deprivation score	−1.40 ± 3.04	−1.34 ± 3.11	−1.45 ± 2.97
Number of long‐term conditions			
0	1444 (35.0)	649 (32.0)	795 (38.0)
1	1329 (32.2)	665 (32.7)	664 (31.8)
2	764 (18.5)	409 (20.1)	355 (17.0)
3	353 (8.7)	183 (9.0)	170 (8.1)
≥4	232 (5.6)	125 (6.2)	107 (5.1)

### Estimated prevalence of low muscle mass

**Figure 2:**
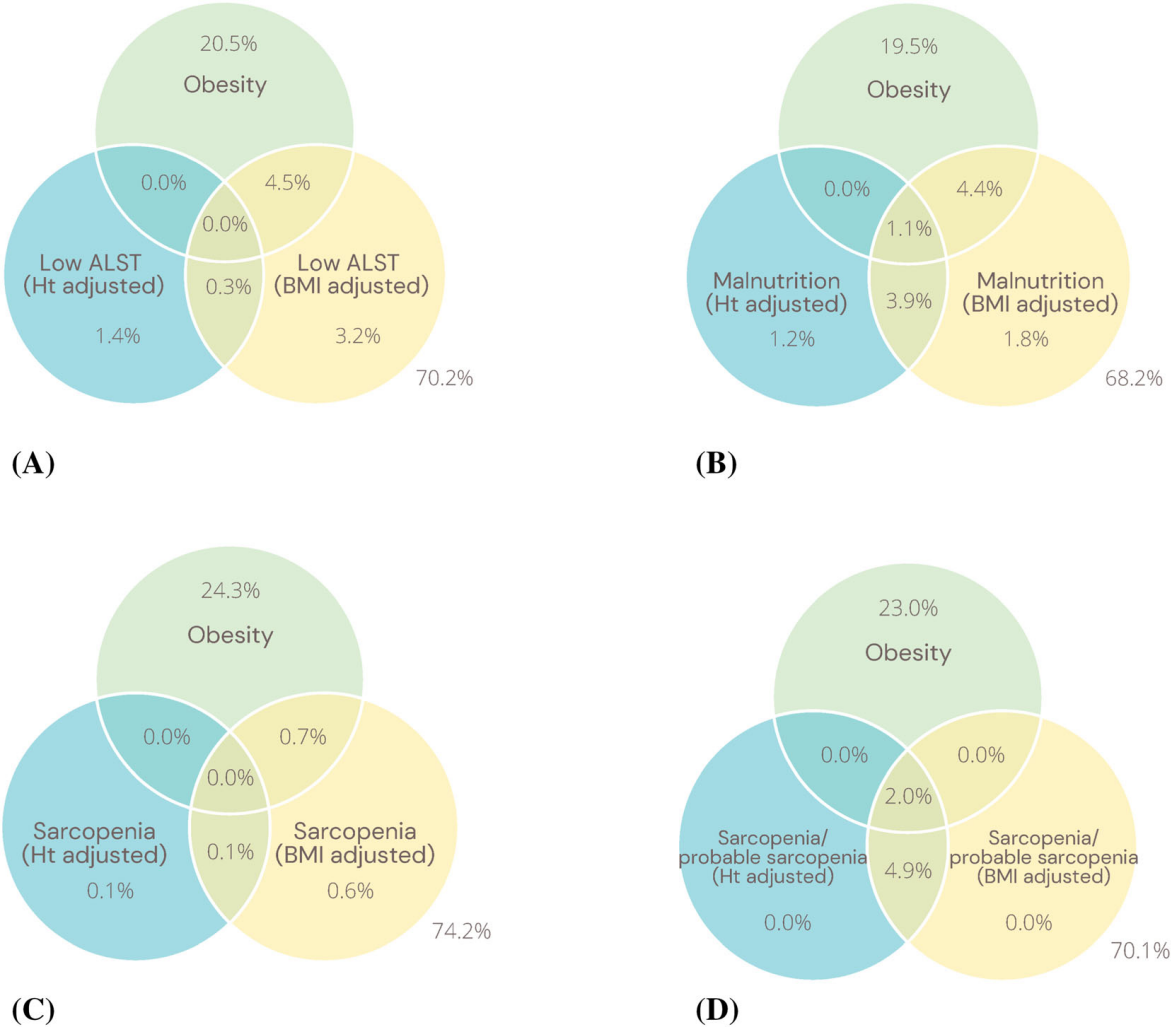
Venn diagrams demonstrating proportion of participants with (A) obesity and low ALST adjusted for height and BMI; (B) obesity and malnutrition when low ALST is adjusted for height and BMI; (C) obesity and sarcopenia when low ALST is adjusted for height and BMI; (D) obesity and sarcopenia/probable sarcopenia when low ALST is adjusted for height and BMI (*N* = 4122). Ht, height; BMI, body mass index; ALST, appendicular lean soft tissue.

Regardless of the adjustment method, low muscle mass was more prevalent in men than women, and in participants aged ≥65 compared with <65 years old (Table 2). The estimated prevalence of low muscle mass was 1.7% using ALST/height^2^, and 8.0% using ALST/BMI (Table 3). Notably, no participants with low ALST/height^2^ had obesity, whereas more than half (56.3%) of participants with low ALST/BMI had obesity (Figure 2A). The highest prevalence of low ALST/height^2^ within individual cancer types was observed in head and neck cancer (11.8%), lung cancer (8.5%), and unknown primary cancers (3.7%). When adjusted for BMI, low muscle mass was most common in bone and soft tissue (16.1%), genitourinary (11.7%), and lung (10.9%) cancers, although numbers were quite small for some cancer types.

**Table 3:** Estimated prevalence of low muscle mass (using ALST), malnutrition, probable sarcopenia and sarcopenia by age and sex (*N* = 4122)

Body composition parameter	Low ALST/height^2^		Low ALST/BMI		Low ALST/height^2^		Low ALST/BMI	
	Men (*n* = 2031)	Women (*n* = 2091)	Men (*n* = 2031)	Women (*n* = 2091)	<65 years (*n* = 2842)	≥65 years (*n* = 1280)	<65 years (*n* = 2842)	≥65 years (*n* = 1280)
	*n* (%)	*n* (%)	*n* (%)	*n* (%)	*n* (%)	*n* (%)	*n* (%)	*n* (%)
Malnutrition								
Malnourished	147 (7.2)	107 (5.1)	221 (10.9)	239 (11.4)	178 (6.3)	76 (5.9)	304 (10.7)	156 (12.2)
Well nourished	1884 (92.8)	1984 (94.9)	1810 (89.1)	1852 (93.9)	2664 (95.7)	1204 (96.1)	2538 (89.3)	1124 (87.8)
Appendicular lean soft tissue								
Low	65 (3.2)	3 (0.1)	234 (11.5)	93 (4.4)	33 (1.2)	35 (2.7)	179 (6.3)	148 (11.5)
Normal	1966 (96.8)	2088 (99.9)	1797 (88.5)	1998 (95.6)	2809 (98.8)	1245 (97.3)	2663 (93.7)	1132 (88.5)
Sarcopenia								
Sarcopenic	9 (0.4)	0 (0)	41 (2.0)	13 (0.6)	4 (0.1)	5 (0.4)	26 (0.9)	28 (2.2)
Probable sarcopenia	130 (6.4)	145 (6.9)	98 (4.8)	132 (6.3)	158 (5.6)	117 (9.1)	136 (4.8)	94 (7.3)
Non‐sarcopenic	1892 (93.2)	1946 (93.1)	1892 (93.2)	1946 (93.1)	2680 (94.3)	1158 (90.5)	2680 (94.3)	1158 (90.5)

ALST, appendicular lean soft tissue.

### Estimated prevalence of malnutrition

**Figure 3:**
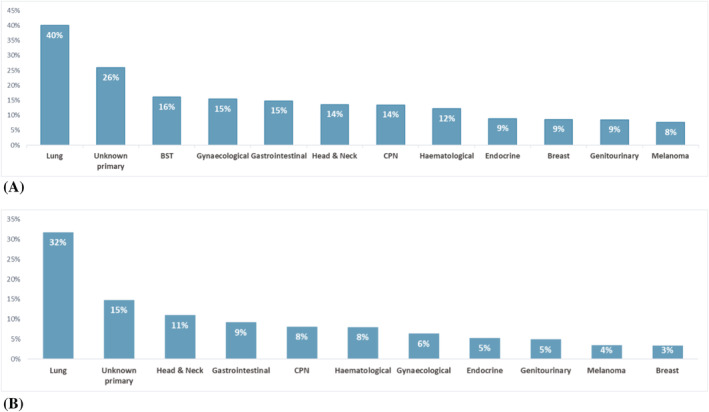
Estimated prevalence of malnutrition by cancer type when (A) muscle mass is adjusted for body mass index and (B) when muscle mass is adjusted for height squared. BST, bone and soft tissue; CPN, central and peripheral nervous system.

The prevalence of malnutrition was similar by sex and in participants aged ≥65 or <65 years (Table 3). Using low ALST/height^2^, the overall estimated prevalence of malnutrition was 6.2%, with 3.9% classified as severely malnourished. Overall, 18.5% of participants with malnutrition using low ALST/height^2^ had obesity, while almost half of participants (49.3%) with malnutrition using low ALST/BMI had obesity (Figure 2B). The cancer types with the highest prevalence of low ALST/height^2^ were lung cancer (31.7%), unknown primary cancers (14.8%) and head and neck cancers (11.0%) (Table 4, Figure 3). However, when low ALST/BMI was used the overall prevalence of malnutrition was 11.2%, with 5.9% classified with severe malnutrition (Table 4). The prevalence of malnutrition was higher for all cancer types using low ALST/BMI, with the highest prevalence of malnutrition in lung (40.0%), unknown primary (25.9%), and bone soft tissue (16.1%) cancers (Figure 3).

**Table 4:** Estimated prevalence of low muscle mass (using ALST) adjusted for height (in m^2^) or body mass index (BMI), and its impact on malnutrition, probable sarcopenia, and sarcopenia prevalence by cancer diagnosis (*N* = 4122)

	Total	Bone and soft tissue (*n* = 31)	Breast (*n* = 1075)	Central and peripheral nervous (*n* = 37)	Endocrine & Thyroid (*n* = 56)	Gastrointestinal (*n* = 607)	Genitourinary (*n* = 1192)	Gynaecological (*n* = 298)	Haematological (*n* = 311)	Head and neck (*n* = 118)	Lung and other thoracic (*n* = 82)	Melanoma (*n* = 288)	Unknown primary (*n* = 27)
	*n* (%)	*n* (%)	*n* (%)	*n* (%)	*n* (%)	*n* (%)	*n* (%)	*n* (%)	*n* (%)	*n* (%)	*n* (%)	*n* (%)	*n* (%)
ALST/height^2^													
Low	68 (1.7)	0 (0)	0 (0)	0 (0)	2 (3.5)	18 (3.0)	22 (1.8)	1 (0.3)	2 (1.9)	14 (11.8)	7 (8.5)	1 (0.3)	1 (3.7)
Normal	4054 (98.3)	31 (100)	1075 (100)	37 (100)	54 (96.5)	589 (97.0)	1170 (98.2)	297 (99.7)	309 (98.1)	104 (88.2)	75 (86.6)	287 (99.7)	26 (96.3)
ALST/BMI													
Low	327 (8.0)	5 (16.1)	45 (4.1)	1 (2.7)	3 (5.3)	56 (9.2)	139 (11.7)	25 (8.4)	19 (6.1)	11 (7.8)	9 (10.9)	12 (4.1)	2 (7.4)
Normal	3795 (92.0)	26 (83.9)	1030 (95.9)	36 (97.3)	53 (94.7)	551 (90.8)	1053 (88.3)	273 (91.6)	292 (95.9)	107 (92.2)	73 (89.1)	276 (95.9)	25 (96.6)
Malnutrition (ALST/height^2^)													
Malnourished	254 (6.2)	0 (0)	37 (3.4)	3 (8.1)	3 (5.3)	56 (9.2)	58 (4.9)	19 (6.4)	25 (8.0)	13 (11.0)	26 (31.7)	10 (3.5)	4 (14.8)
Severely malnourished^a^	163 (3.9)	0 (0)	28 (2.6)	2 (5.4)	3 (5.3)	39 (6.4)	23 (1.9)	16 (5.4)	18 (5.8)	11 (9.3)	13 (15.8)	6 (2.1)	4 (14.8)
Well nourished	3868 (93.8)	31 (100)	1038 (96.6)	34 (91.9)	53 (94.7)	551 (90.8)	1134 (95.1)	279 (93.6)	286 (92.0)	105 (89.0)	56 (68.3)	278 (96.5)	23 (85.2)
Malnutrition (ALST/BMI)													
Malnourished	460 (11.2)	5 (16.1)	92 (8.6)	5 (13.5)	5 (8.9)	90 (14.8)	101 (8.5)	46 (15.4)	38 (12.2)	16 (13.6)	33 (40.0)	22 (7.6)	7 (25.9)
Severely malnourished^a^	243 (5.9)	2 (6.4)	44 (4.1)	2 (5.4)	2 (1.8)	54 (8.9)	48 (4.0)	28 (9.4)	21 (6.7)	13 (11.0)	16 (19.5)	8 (2.8)	5 (18.5)
Well nourished	3662 (88.8)	26 (83.9)	983 (91.4)	32 (86.5)	51 (91.1)	517 (85.2)	1091 (91.5)	252 (84.6)	273 (87.8)	102 (86.4)	49 (60.0)	266 (92.4)	20 (74.1)
Sarcopenia (ALST/height^2^)													
Sarcopenia	9 (0.2)	0 (0)	(0)	0 (0)	0 (0)	2 (0.3)	3 (0.3)	0 (0)	1 (0.3)	1 (0.8)	2 (2.4)	0 (0)	0 (0)
Probable sarcopenia	275 (6.7)	1 (3.2)	78 (7.3)	3 (8.1)	0 (0)	44 (7.3)	90 (7.5)	18 (6.1)	19 (6.1)	7 (6.0)	4 (4.8)	10 (3.5)	1 (3.7)
Non‐sarcopenic	3838 (93.1)	30 (96.8)	997 (92.7)	34 (91.9)	56 (100)	561 (92.4)	1099 (92.2)	280 (94.0)	291 (93.6)	110 (93.2)	76 (92.8)	278 (96.5)	26 (96.3)
Sarcopenia (ALST/BMI)													
Sarcopenic	54 (1.4)	0 (0)	7 (0.6)	1 (2.7)	0 (0)	7 (1.2)	28 (2.3)	3 (1.0)	2 (0.6)	1 (0.8)	3 (3.6)	1 (0.3)	1 (3.7)
Probable sarcopenia	230 (5.5)	1 (3.2)	71 (6.7)	2 (5.4)	0 (0)	39 (6.4)	65 (5.5)	15 (5.0)	18 (5.8)	7 (6.0)	3 (3.6)	9 (3.2)	0 (0)
Non‐sarcopenic	3838 (93.1)	30 (96.8)	997 (92.7)	34 (91.9)	56 (100)	561 (92.4)	1099 (92.2)	280 (94.0)	291 (93.6)	110 (93.2)	76 (92.8)	278 (96.5)	26 (96.3)

ALST, appendicular lean soft tissue.

^a^ Severely malnourished is a subset of malnourished.

### Estimated prevalence of probable sarcopenia and sarcopenia

Table 4 reports the estimated prevalence of sarcopenia based on low ALST/height^2^, with sarcopenia observed in 0.2% of participants, and being most common in lung (2.4%) and head and neck (0.8%) cancers. Of the nine participants with sarcopenia, five had severe sarcopenia. No participants with sarcopenia using low ALST/height^2^ had obesity, compared with 50% using low ALST/BMI who had both sarcopenia and obesity (Figure 2C). The prevalence of probable sarcopenia (low grip strength) was 6.7%, with central and peripheral nervous system (8.1%), genitourinary (7.5%), and gastrointestinal and breast (both 7.3%) cancers having the highest prevalence. Both sarcopenia and probable sarcopenia had a similar prevalence by sex (Table 3), and both had a slightly higher prevalence in participants aged ≥65 years (Table 3).

Using ALST/BMI (Table 4), the overall estimated prevalence of sarcopenia was 1.4%, and the diagnoses with the highest prevalence were unknown primary (3.7%), lung (3.6%) and central and peripheral nervous system (2.7%) cancers. Of the 54 participants with sarcopenia, 20 had severe sarcopenia. The prevalence of probable sarcopenia (low grip strength) was lower at 5.5%, and among individual cancer types the highest prevalence was seen in breast (6.7%), gastrointestinal (6.4%) and head and neck (6.0%) cancers. Sarcopenia was more prevalent in men (2.0%) than women (0.6%), while the reverse occurred for probable sarcopenia (Table 3). When prevalence was determined by age, participants ≥65 years old had a higher prevalence of both sarcopenia (2.2% vs. 0.9%) and probable sarcopenia (7.3% vs. 4.0%) (Table 3).

### Overlap between conditions based on adjustment of low muscle mass: height^2^ versus body mass index

The overlap in estimated prevalence of low muscle mass, and each condition, when ALST/height^2^ was used compared with ALST/BMI is presented in Figure 2. Overall, while 1.7% of participants had low muscle mass using ALST/height^2^ and 8% of participants had low muscle mass using ALST/BMI, only 0.3% of the whole sample were classified with low muscle mass by both methods (Figure 2A). Regarding malnutrition, the prevalence was 6.2% using ALST/height^2^ and 11.2% using ALST/BMI, with 5.0% classified as malnourished using both adjustments (Figure 2B). Figure 2C shows that sarcopenia using ALST/height^2^ was present in 0.2% and 1.4% using ALST/BMI, with only 0.1% of the whole sample classified as sarcopenic using both methods. When the prevalence of sarcopenia and probable sarcopenia were combined (Figure 2D), there was complete overlap between the ALST/height^2^ and ALST/BMI methods and the proportion of these participants who had concurrent obesity (i.e., sarcopenic obesity).

### Association with all‐cause and cancer‐specific mortality

**Figure 4:**
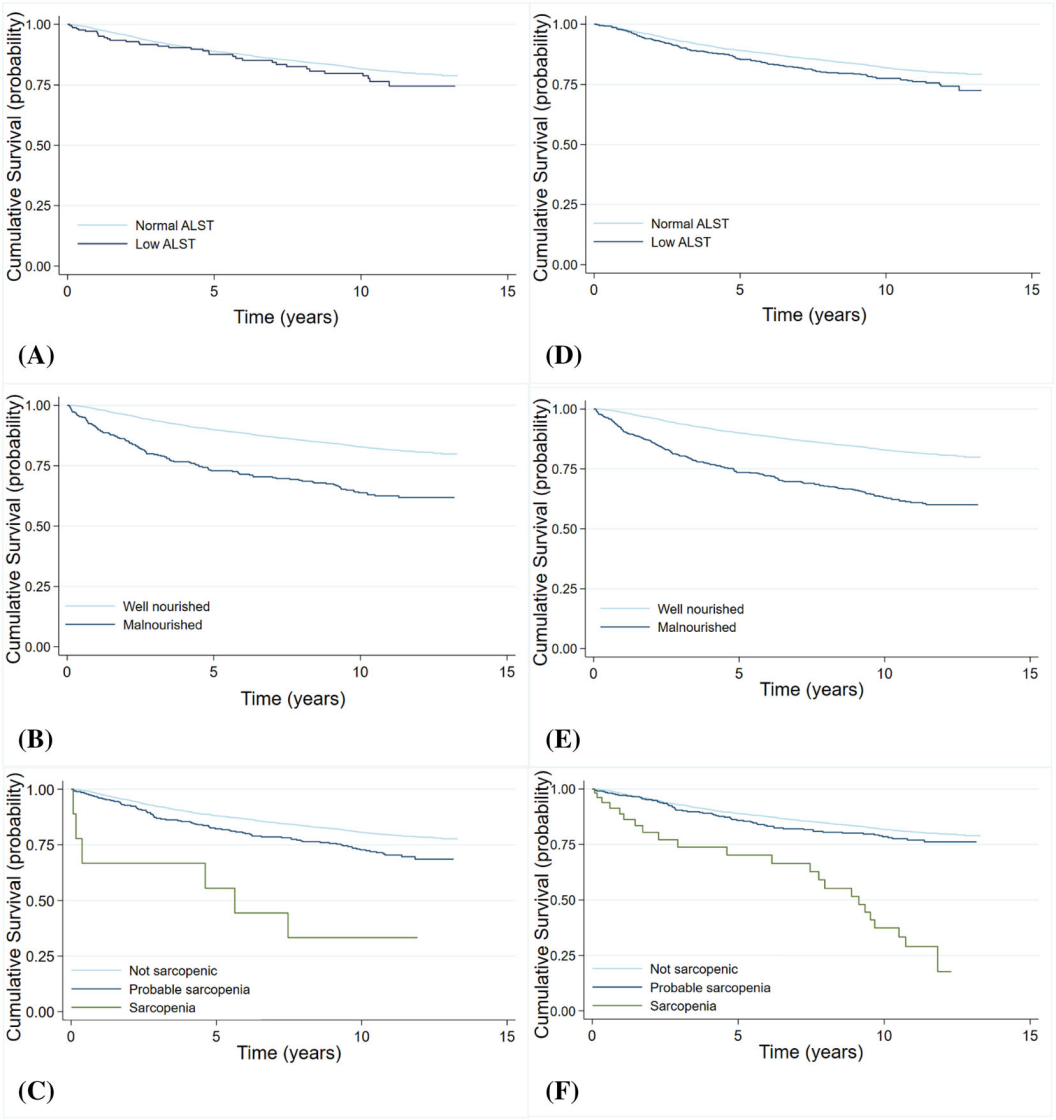
Covariate‐adjusted Kaplan–Meier overall survival curves for (A) ALST adjusted for height, (B) malnutrition using ALST adjusted for height, (C) sarcopenia using ALST adjusted for height (unadjusted for coavriates), d) ALST adjusted for BMI, (E) malnutrition using ALST adjusted for BMI, and (F) sarcopenia using ALST adjusted for BMI (*N* = 4122). ALST, appendicular lean soft tissue; BMI, body mass index. The curves were estimated based on the mean level of the covariates.

During a median 11.2 (interquartile range: 10.2, 12.0) years of follow up, 901 (21.7%) of the 4122 participants died, and of these, 744 (82.6%) deaths were cancer‐specific. Low muscle mass, malnutrition (analysed as both severe vs. not severe, and malnourished vs. well nourished), and sarcopenia were all associated with a higher relative risk of all‐cause and cancer‐specific mortality, regardless of whether muscle mass was adjusted for height or BMI (Table 5). There was moderate evidence that probable sarcopenia (low grip strength) was associated with a higher risk of all‐cause mortality, using either adjustment. There was insufficient evidence of an association between probable sarcopenia and cancer‐specific mortality. Covariate‐adjusted Kaplan–Meier curves are presented for overall (all‐cause) survival (Figure 4) and cancer‐specific survival (Figure S1).

**Table 5:** Association between low muscle mass (using ALST), malnutrition, probable sarcopenia, and sarcopenia with all‐cause and cancer‐specific mortality (*N* = 4122)

Condition	All‐cause mortality^a^			Cancer‐specific mortality^a^		
	Death (*n*/*N*)	Hazard ratio (95% CI)	*P*‐value	Death (*n*/*N*)	Hazard ratio (95% CI)	*P*‐value
ALST/height^2^						
Low	35/68	1.9 (1.3, 2.8)	0.001	29/68	2.0 (1.3, 3.2)	0.001
Normal	859/4054	1.0 (reference)		711/4054	1.0 (reference)	
ALST/BMI						
Low	108/327	1.4 (1.1, 1.7)	0.002	84/327	1.4 (1.1, 1.8)	0.006
Normal	786/3795	1.0 (reference)		656/3795	1.0 (reference)	
Malnutrition (ALST/height^2^)						
Severe malnutrition	78/163	2.8 (2.2, 3.6)	<0.001	65/163	2.9 (2.2, 3.7)	<0.0005
Not severe malnutrition	816/3959	1.0 (reference)		675/3959	1.0 (reference)	
Malnourished^b^	118/254	2.5 (2.0, 3.1)	<0.001	97/254	2.6 (2.1, 3.2)	<0.0005
Well nourished	776/3868	1.0 (reference)		643/3868	1.0 (reference)	
Malnutrition (ALST/BMI)						
Severe malnutrition	109/243	2.7 (2.2, 3.4)	<0.001	88/243	2.7 (2.1, 3.4)	<0.0005
Not severe malnutrition	785/3879	1.0 (reference)		652/3879	1.0 (reference)	
Malnourished	176/460	2.2 (1.8, 2.6)	<0.001	145/460	2.3 (1.9, 2.7)	<0.0005
Well nourished	718/3662	1.0 (reference)		595/3662	1.0 (reference)	
Sarcopenia (ALST/height^2^)						
Sarcopenic	6/9	2.9 (1.3, 6.5)	0.013	6/9	3.6 (1.6, 8.2)	0.003
Probable sarcopenia	82/275	1.3 (1.0, 1.6)	0.022	62/275	1.2 (0.9, 1.6)	0.128
Non‐sarcopenic	806/3838	1.0 (reference)		672/3838	1.0 (reference)	
Sarcopenia (ALST/BMI)						
Sarcopenic	23/54	1.6 (1.0, 2.4)	0.032	16/54	1.5 (0.9, 1.7)	0.136
Probable sarcopenia	65/230	1.3 (1.0, 1.7)	0.048	52/230	1.5 (0.9, 2.4)	0.113
Non‐sarcopenic	806/3838	1.0 (reference)		672/3838	1.0 (reference)	

ALST, appendicular lean soft tissue.

^a^ Adjusted for BMI (height‐adjusted measures only), age, sex, time since cancer diagnosis, smoking status, alcohol intake, and number of co‐morbidities.

^b^ Malnutrition includes both mild‐moderate and severe malnutrition.

#### Comparing low muscle mass by method of adjustment: height^2^ versus body mass index

Low ALST/height^2^ was more strongly associated with both all‐cause (HR: 1.9 vs. HR: 1.4) and cancer‐specific (HR: 2.0 vs. HR: 1.4) mortality than low ALST/BMI. In contrast, for malnutrition the relative hazard of both all‐cause and cancer‐specific mortality were comparable regardless of the adjustment method, with malnutrition consistently associated with more than two times higher hazard, and a higher hazard seen with increasing malnutrition status (Table 5). When ALST/height^2^ was used for sarcopenia, the hazard ratio of both all‐cause (HR: 2.9 vs. HR: 1.6) and cancer‐specific (HR: 3.6 vs. HR: 1.5) mortality were greater than when ALST/BMI was used.

## Discussion

In this study of 4122 UK Biobank adults within 2 years of a cancer diagnosis, we found low muscle mass, malnutrition, and sarcopenia were associated with a 1.4 to 3.6 times higher risk for all‐cause mortality. The relative hazard for mortality increased with malnutrition severity. Malnutrition was more prevalent than both low muscle mass and sarcopenia, regardless of the method of muscle mass adjustment for body size. However, when muscle mass was adjusted for BMI compared with height, a higher estimated prevalence of low muscle mass, malnutrition, and sarcopenia was observed. Furthermore, BMI adjustment of muscle mass identified more cases of low muscle mass, malnutrition, and sarcopenia in participants with obesity. Finally, with the exception of malnutrition, the overlap in prevalence of low muscle mass and sarcopenia between the methods of adjustment was relatively low, indicating each method was identifying different participants with low muscle mass/sarcopenia.

The prevalence of low muscle mass (1.7–7.9%), malnutrition (6.2–11.2%), and sarcopenia (0.2–1.3%) using different methods of muscle mass adjustment for body size in our study of people with cancer appeared low, although it is difficult to compare to previous studies in cancer populations as these have typically used different techniques to determine low muscle mass. Two previous studies in mixed newly diagnosed cancer populations reported a prevalence of low muscle mass ranging from 15 to 19.2% based on low fat‐free mass index (FFMI, kg/m^2^) derived from BIA.^17^, ^30^ This is higher than the prevalence of low muscle mass based on low ALST used in our study, potentially due to the aforementioned studies including predominantly people with gastrointestinal, head and neck and lung cancers as opposed to the predominantly breast and genitourinary cancers in our study. Differences in results may also be explained by BIA limitations in this population and the inclusion of participants with metastatic disease because metastasis are included when fat‐free mass is assessed.^31^ Malnutrition prevalence as high as 77%, using the GLIM criteria with low muscle mass determined by FFMI, has been reported in previous cancer studies, albeit in people with advanced cancer.^32^, ^33^ Two studies, which assessed sarcopenia using CT‐derived skeletal muscle index (SMI cm^2^/m^2^), low grip strength and low gait speed found a prevalence of sarcopenia of 11.9% and 12.5% in post‐surgical patients with colorectal and gastric cancer, respectively.^34^, ^35^ A cross‐sectional study of 70 men with prostate cancer applied the EWGSOP2 sarcopenia definition using DXA‐derived ALST/height^2^ and found no participants had sarcopenia.^36^ To our knowledge, no previous studies have estimated GLIM defined malnutrition or EWGSOP2 defined sarcopenia using a BMI‐adjusted measure of low muscle mass in people with cancer. The relatively low prevalence of low muscle mass, malnutrition, and sarcopenia in our study is likely to reflect the mix of cancer types, of which breast and genitourinary cancers comprised more than half the sample. Furthermore, participants in our study were on average just over a year post‐cancer diagnosis and are likely to represent people at various stages of recovery from a broad range of treatments, and varying disease stage, factors that were not available in the UK Biobank at the time of analysis. Finally, the EWGSOP2 is a criterion for primary sarcopenia (i.e., age‐related), and most participants were not older adults. Our findings highlight the potential limitation of these criteria in the context of secondary sarcopenia (i.e., disease related).^4^

An important finding from our study was that regardless of whether low muscle mass was adjusted for height or BMI, the presence of low muscle mass, malnutrition, and sarcopenia was associated with an increased risk of all‐cause mortality. This is consistent with previous research using CT and BIA measures of muscle mass,^2^, ^5^, ^16^, ^17^ although to our knowledge this is the first study to demonstrate an association with mortality using a BMI‐adjusted measure of muscle mass in a cancer population. We also observed a similar association for cancer‐specific mortality, with the exception of probable sarcopenia and sarcopenia, for which there was limited evidence of increased risk of cancer‐specific mortality potentially due to reduced power with the combination of a relatively small number of cancer‐specific deaths and cases of (probable)sarcopenia. In terms of risk stratification, our findings indicate that participants with increasing severity of malnutrition are at greatest risk of mortality and require prioritization for intervention. However, previous research shows there are adverse outcomes other than survival that are associated with low muscle mass and malnutrition, such as reduced tolerance to and completion of treatment.^2^, ^37^ These outcomes are not available within the UK Biobank and require further investigation using height‐ and BMI‐adjusted measures.

In our UK Biobank sample of adults with cancer, in which 25% of participants had obesity, only the BMI‐adjusted method identified cases of low muscle mass and sarcopenia in participants with obesity. This suggests that low muscle mass adjusted for height may underestimate the prevalence in people with obesity. Furthermore, when using the BMI adjustment approximately half of the cases of low muscle mass, malnutrition, and sarcopenia were in people with concurrent obesity. This may be related to the BMI adjustment taking into consideration overall body mass, and not exclusively stature.^15^ Heymsfield and colleagues' findings showed the inadequacy of using skeletal muscle index (SMI cm^2^/m^2^) to identify low muscle mass in people with a higher BMI,^15^ which is also common in patients with cancer, despite a high prevalence of low muscle mass and malnutrition.^38^ Sarcopenic obesity, the simultaneous occurrence of sarcopenia or low muscle mass and obesity, is a body composition phenotype independently associated with higher mortality and increased treatment complications in people with cancer.^6^ Therefore, the ability to detect low muscle mass in people with obesity is crucial to facilitate timely referral for intervention to reduce the risk of adverse outcomes. Cases of malnutrition were identified in participants with obesity using the height‐adjusted method, although a greater number of cases were identified using the BMI adjustment. This is most likely due to the complexity of the malnutrition diagnosis, where low muscle mass was only one of three phenotypic criteria considered for the diagnosis of malnutrition, and hence the diagnosis was less reliant on low muscle mass being present.^8^

Another important finding from our study was that there was minimal overlap between low muscle mass or sarcopenia identified using BMI compared with the height‐adjusted method, meaning each method was identifying different participants as having low muscle mass and sarcopenia. When sarcopenia and probable sarcopenia (low grip strength alone) were combined there was complete overlap in prevalence of combined sarcopenia/probable sarcopenia using BMI‐ and height‐adjusted low muscle mass as this represents all participants with low grip strength regardless of whether they had low muscle mass. In contrast, there was a greater degree of overlap between malnutrition using BMI‐adjusted compared with height‐adjusted muscle mass. As discussed earlier, this difference is most likely related to the greater complexity of the malnutrition diagnosis, which may be diagnosed in the absence of low muscle mass due to the presence of weight loss. Nevertheless, our findings suggest that BMI‐adjusted muscle mass may be a better method of identifying low muscle mass, malnutrition, and sarcopenia in people with obesity. However, the mortality hazard for each of these conditions was higher when using height‐adjusted compared with BMI‐adjusted muscle mass. This may be explained by the U‐shaped relationship, which has been demonstrated between BMI and mortality, where the lowest risk is observed in the BMI range 25 to <30 kg/m^2^, suggesting some degree of adiposity is protective.^39^

Strengths of this study include the large sample size, the broad range of cancer diagnoses and wide age range of participants. Data available from the UK Biobank allowed adjustment for important sociodemographic lifestyle factors in our analysis, including smoking status, alcohol intake and co‐morbidities. However, the study has some limitations. UK Biobank participants are predominantly white and less socioeconomically deprived than the general population.^40^ It has also been reported there is evidence of healthy volunteer selection bias, meaning our findings may not be representative of the general cancer population.^40^ The estimates of prevalence of outcomes may therefore be underestimated in comparison to other cancer populations. Data available for weight loss, used for the malnutrition diagnosis, was not quantifiable and did not specify if weight loss was intentional or unintentional. Therefore, the prevalence of malnutrition may be overestimated while the effect of malnutrition on mortality may be underestimated. Furthermore, low ALST was estimated from bioelectrical impedance measurement, which has known limitations and then derived using a prediction equation developed in a non‐cancer population, which has potential to introduce error. Cancer stage and prior cancer treatment were not available and therefore could not be accounted for in analysis. The inclusion of participants within a two‐year timeframe of cancer diagnosis is also likely to result in a diverse participant group at varying lengths of time post‐cancer treatment.

In summary, in this cohort of UK Biobank participants with cancer, malnutrition was more prevalent than low muscle mass and sarcopenia; however, all were associated with a higher mortality hazard, regardless of the method of adjusting muscle mass. Adjustment of low muscle mass for BMI identified more cases of low muscle mass, malnutrition, and sarcopenia overall and in participants with obesity compared with height, suggesting it is the preferred adjustment in people with obesity. However, a small number of participants were only identified as having low muscle mass or sarcopenia when low muscle mass was adjusted for height. As these participants were also at higher risk of mortality, in practice using both methods of adjustment would be prudent if feasible. Comparing the association of low muscle mass, malnutrition, and sarcopenia, using these two methods of adjustment with other important outcomes, such as treatment tolerance should be investigated for a broader perspective.

## Conflict of interest statement

C.M.P. reports receiving honoraria and/or paid consultancy from Abbott Nutrition, Nutricia, Nestle Health Science, Fresenius Kabi, Pfizer, and AMRA Medical. Other authors have no conflicts of interest to report.

## Supporting information


**Table S1:** Adaptation of malnutrition, low muscle mass, and sarcopenia criteria for the UK Biobank
**Figure S1:** Covariate adjusted Kaplan Meier cancer‐specific survival curves for a) ALST adjusted for height, b) malnutrition using ALST adjusted for height, c) sarcopenia using ALST adjusted for height (unadjusted for covariates), d) ALST adjusted for BMI, e) malnutrition using ALST adjusted for BMI, and f) sarcopenia using ALST adjusted for BMI (*N* = 4122). ALST, appendicular lean soft tissue; BMI, body mass index. The curves were estimated based on the mean level of the covariates.
