# Supplementary material for: Low muscle mass, malnutrition, sarcopenia, and associations with survival in adults with cancer in the UK Biobank cohort
Source: J Cachexia Sarcopenia Muscle. 2023 May 22;14(4):1775–88. doi: 10.1002/jcsm.13256 (PMC10401543; doi:10.1002/jcsm.13256)
Supplement: Supplementary file 1 — Table S1: Adaptation of malnutrition, low muscle mass, and sarcopenia criteria for the UK Biobank Figure S1: Covariate adjusted Kaplan Meier cancer‐specific survival curves for a) ALST adjusted for height, b) malnutrition using ALST adjusted for height, c) sarcopenia using ALST adjusted for height (unadjusted for covariates), d) ALST adjusted for BMI, e) malnutrition using ALST adjusted for BMI, and f) sarcopenia using ALST adjusted for BMI (N = 4122). ALST, appendicular lean soft tissue; BMI, body mass index. The curves were estimated based on the mean level of the covariates. [file JCSM-14-1775-s001.docx]

**Supplementary material**

Supplementary Table 1: Adaptation of malnutrition, low muscle mass, and sarcopenia criteria for the UK Biobank

| **Component** | **Original criteria** | **UK Biobank** |
| --- | --- | --- |
| ***Malnutrition^a^*** |  |  |
| Weight loss | >5% in past 6 months or >10% beyond 6 months | Self-reported: ‘Compared with one year ago, has your weight changed?’  Response: yes, lost weight = 1 or other = 0 (no, gained weight, do not know) |
| Low BMI | *Mild to moderate:* <20 kg/m^2^ if < 70 years or <22 kg/m^2^ if > 70 years  *Severe:* <18.5 kg/m^2^ if <70 years or <20 kg/m^2^ if > 70 years | *Mild to moderate:* <20 kg/m^2^ if < 70 years or <22 kg/m^2^ if > 70 years  *Severe:* <18.5 kg/m^2^ if <70 years or <20 kg/m^2^ if > 70 years |
| Low muscle mass | *Mild to moderate* deficit by validated assessment methods  *Severe* deficit by validated assessment methods | *Mild to moderate:* ALST^b^/ht^2^ <6.15 kg/m^2^ (female) and <7.98 kg/m^2^ (male) OR ALST/BMI^c^ < 0.64 (females) and <0.94 (males)  *Severe:* ALST/ht^2^ <5.30kg/m^2^ (female) and <6.95kg/m^2^ (male) OR ALST/BMI < 0.55 (females) and <0.84 (males) |
| Inflammation | Acute disease/ injury or chronic-disease related | CRP^d^ >5 mg/L |
| ***Sarcopenia^e^*** |  |  |
| Low muscle strength | Handgrip strength <27 kg (males) and <16 kg (females) | Handgrip strength <27 kg (males) and <16 kg (females) |
| Low muscle mass | ALST/ht^2^ < 7.0 kg/m^2^ (males) or <5.5 kg/m^2^ (females) | ALST/ht^2^ <5.30kg/m^2^ (female) and <6.95kg/m^2^ (male) OR ALST/BMI < 0.55 (females) and <0.84 (males) |
| Low physical performance | Gait speed < 0.8 metres/second | Self-reported: ‘How would you describe your usual walking pace?’  Response: slow = 1, other = 0 (average, brisk, none of the above) |

^a^Assessed using the Global Leadership Initiative on Malnutrition (GLIM) criteria; ^b^ALST, appendicular lean soft tissue; ^c^Body Mass Index; ^d^CRP, C-reactive protein; ^e^ Assessed using the European Working Group on Sarcopenia in Older People 2 (EWGSOP2) definition. Sex-specific cut-points for low ALST/height^2^ and low ALST/BMI specific to the UK Biobank cohort were derived from participants aged 45 years or less as the reference, based on two standard deviations below the sex-specific mean [1].


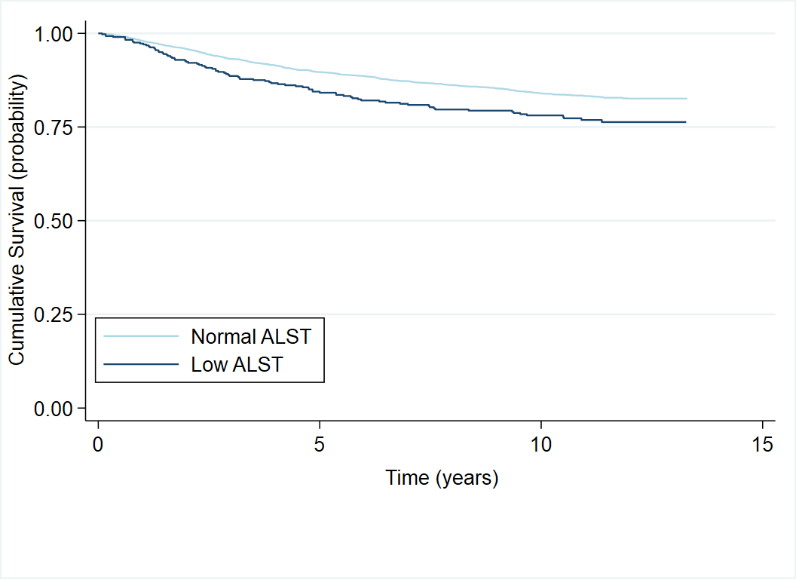

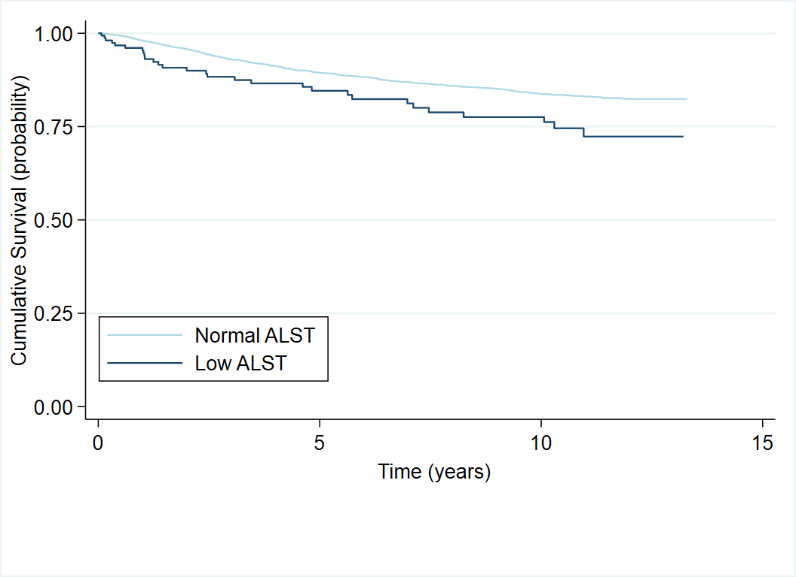


d)

a)


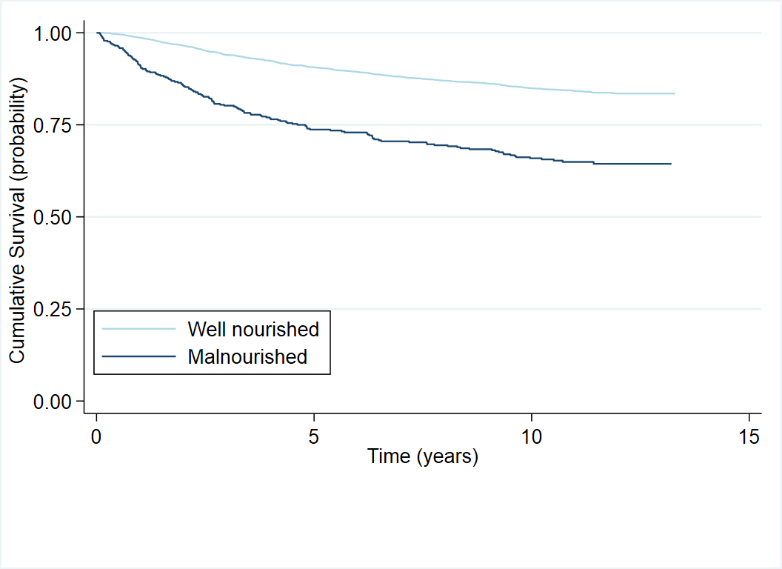

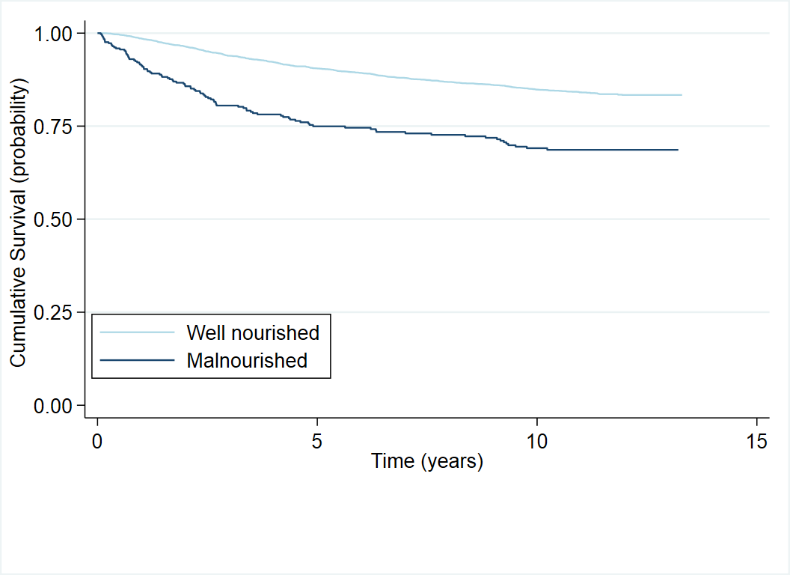


e)

b)


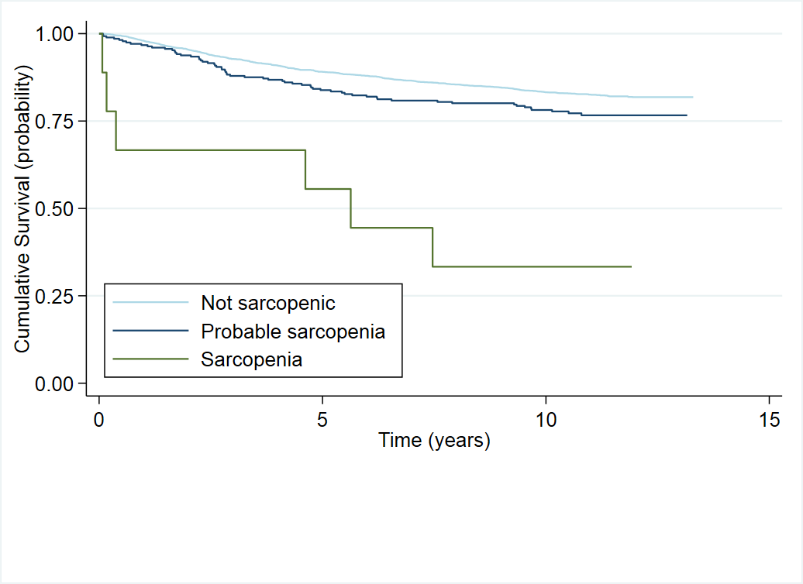

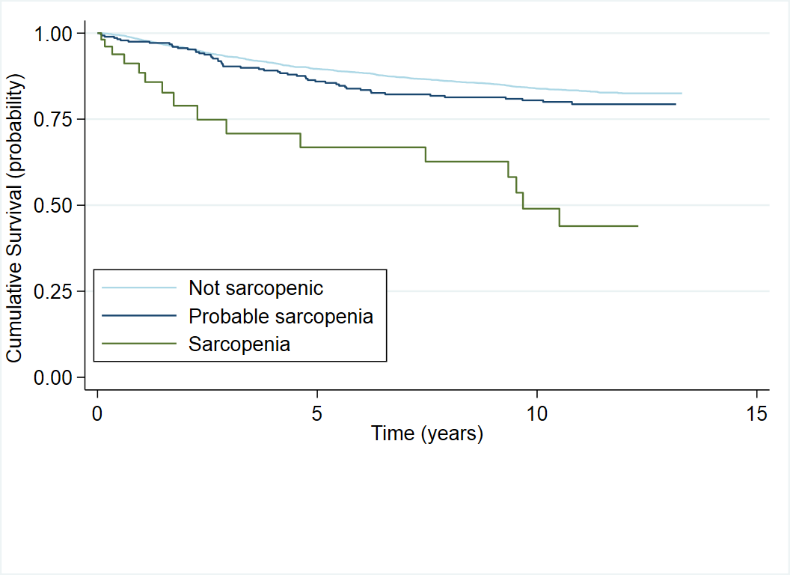


c)

f)


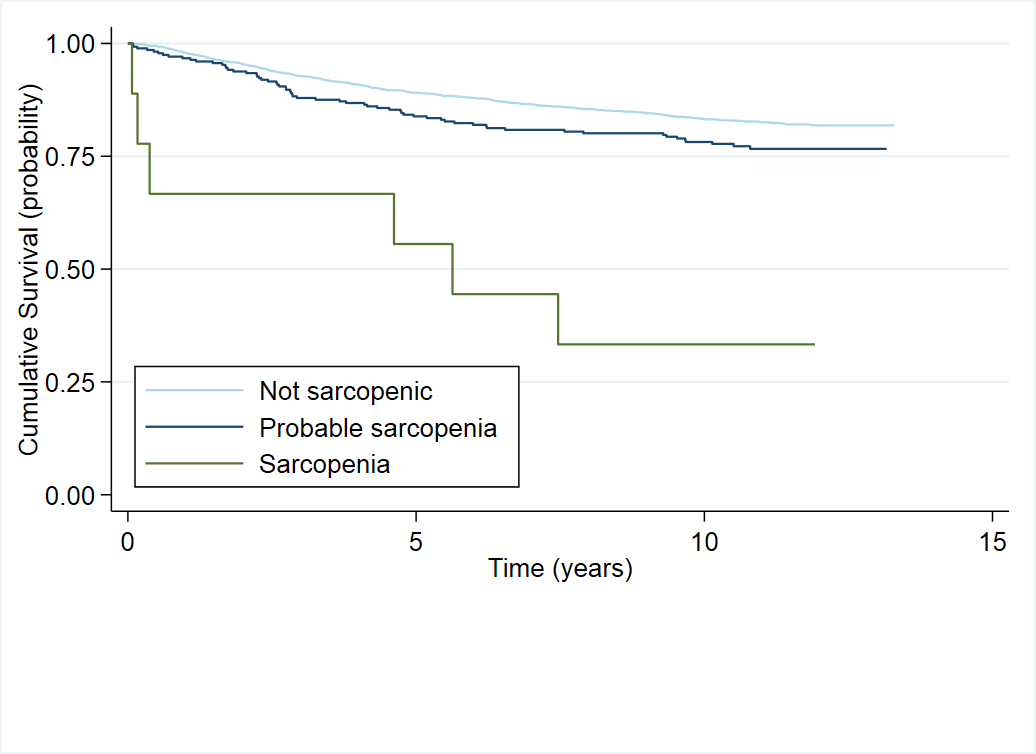


Supplementary Figure 1: Covariate adjusted Kaplan Meier cancer-specific survival curves for a) ALST adjusted for height, b) malnutrition using ALST adjusted for height, c) sarcopenia using ALST adjusted for height (unadjusted for covariates), d) ALST adjusted for BMI, e) malnutrition using ALST adjusted for BMI, and f) sarcopenia using ALST adjusted for BMI (N= 4122). ALST, appendicular lean soft tissue; BMI, body mass index. The curves were estimated based on the mean level of the covariates.

References:

1. Gould H, Brennan S.L, Kotowicz M.A, Nicholson G.C, Pasco J.A. Total and appendicular lean mass reference ranges for Australian men and women: the Geelong osteoporosis study. Calcif Tissue Int. 2014;94(4):363-72.
